# Analyzing the Performance of ChatGPT About Osteoporosis

**DOI:** 10.7759/cureus.45890

**Published:** 2023-09-25

**Authors:** Cigdem Cinar

**Affiliations:** 1 Department of Interventional Physiatry, Biruni University, Istanbul, TUR

**Keywords:** information source, osteoporosis, guideline, chatgpt, artificial intelligence

## Abstract

Introduction: This study evaluates the knowledge of ChatGPT about osteoporosis.

Methods: Osteoporosis-related frequently asked questions (FAQs) created by examining the websites frequently visited by patients, the official websites of hospitals, and social media. Questions based on these scientific data have been prepared in accordance with National Osteoporosis Guideline Group guides. Rater scored all ChatGPT answers between 1 and 4 (1 stated that the information was completely correct, 2 stated that the information was correct but insufficient, 3 stated that although some of the information was correct, there was incorrect information in the answer, and 4 stated that the answer consisted of completely incorrect information). The reproducibility of ChatGPT responses on osteoporosis was assessed by asking each question twice. The repeatability of the ChatGPT answer was considered as getting the same score twice.

Results: ChatGPT responded to 72 FAQs with an accuracy rate of 80.6%. The highest accuracy in ChatGPT's answers about osteoporosis was in the prevention category, 91.7%, and in the general knowledge category, 85.8%. Only 19 of the 31 (61.3%) questions prepared according to the National Osteoporosis Guideline Group guidelines were answered correctly by ChatGPT, and two answers (6.4%) were categorized as grade 4. The reproducibility rate of ChatGPT answers on 72 FAQs was 86.1% and the reproducibility rate of ChatGPT answers on National Osteoporosis Guideline Group guidelines was 83.9%.

Conclusion: Present study outcomes for the first time showed that ChatGPT provided adequate answers to more than 80% of FAQs about osteoporosis. However, the accuracy of ChatGPT’s answers to inquiries based on National Osteoporosis Guideline Group guidelines was decreased to 61.3%.

## Introduction

Osteoporosis is characterized by reduced bone mass, bone tissue deterioration, and pathological changes in bone microarchitecture, which result in a decrease in bone strength and an increase in the risk of bone fractures [1]. Osteoporosis has an economic, medical, and social burden, and previous reports emphasized the relation between osteoporosis and prolonged and sedentary lifestyles, economic deficiencies, alcohol use, endocrine diseases, and malign disorders [2]. Salari et al. reviewed 70 studies including 800,457 women and 453,964 men, and study findings stated that 11.7% of men and 23.1% of women had osteoporosis [3]. On the other hand, new-generation treatment modalities have been developed for osteoporosis, and numerous studies about osteoporosis demonstrated that patients’ awareness and knowledge about osteoporosis are crucial in the prevention and treatment of osteoporosis [2,4]. In recent years, web sources have an indispensable role in raising awareness of public health, and a large number of patients get information about their health problems from online sources including Instagram, Twitter, YouTube, and generative pre-trained transformer (ChatGPT) [5].

ChatGPT, which was created by OpenAI, is an artificial intelligence application with natural language programming. While ChatGPT can be used in all areas of life, recent studies have been investigating the effectiveness and reliability of using ChatGPT in the field of health [6]. Caglar et al. analyzed the precision accuracy and consistency of ChatGPT's responses to questions in pediatric urology, and authors achieved satisfactory answers to questions related to pediatric urology [7]. Also, Gilson et al. analyzed the performance of ChatGPT in medical school exams, and ChatGPT gave correct answers to 60% of the questions [8]. Moreover, Rao et al. stated that ChatGPT had a satisfactory accuracy rate in the evaluation of radiological findings [9].

Although previous studies investigated the accuracy of ChatGPT answers in various disorders, to our knowledge, no research has analyzed the precision and consistency of ChatGPT's responses to osteoporosis. The aim of this study was to evaluate the knowledge of ChatGPT about osteoporosis.

## Materials and methods

Osteoporosis-related frequently asked questions (FAQ) were created by examining the websites frequently visited by patients and the official websites of hospitals. Websites affiliated with authoritative and reputable organizations were given preference. These organizations often include government health agencies, leading medical institutions, academic research centers, and recognized osteoporosis advocacy groups. In addition, the questions asked by patients on social media platforms such as YouTube, Facebook, and Instagram and patient comments on these platforms were used while preparing the question list (Table 3, Appendix). Scientific data and questions based on these scientific data have been prepared in accordance with National Osteoporosis Guideline Group (NOGG) guides, and all these questions were collected in a separate questionnaire (Table 4, Appendix). Unrealistic questions, repetitive questions, questions containing advertisements, grammatically incorrect questions, and questions requiring personal answers were excluded from the study. All questions in FAQ form classified as general information (n = 14), risk factors (n = 10), non-pharmacological treatments (n = 12), pharmacological treatments (n = 24), and prevention (n = 12). Questions depended on NOGG guidelines and included 31 questions.

The answers were evaluated by a physiotherapist with eight years of clinical and academic experience in osteoporosis. Rater scored all ChatGPT answers between 1 and 4 (1 stated that the information was completely correct, 2 stated that the information was correct but insufficient, 3 stated that although some of the information was correct, there was incorrect information in the answer, and 4 stated that the answer consisted of completely incorrect information). The definition of the correct answer is an experienced physiotherapist was considered to have made no additional contribution to the ChatGPT response to a patient question. In the questions related to the NOGG guides, guideline information was taken into account when evaluating the accuracy of ChatGPT's answers.

The reproducibility of ChatGPT responses on osteoporosis was assessed by asking each question twice in written form on different days. The repeatability of the ChatGPT answer was considered as getting the same score twice. Two ChatGPT answers in different score categories or having different levels of detail were evaluated negatively in terms of reproducibility. Ethics committee approval was not required as patient data was not used in the present study.

Statistical analysis

Statistical analysis was performed using Excel Version 16 (Microsoft Corporation, USA). The questions were evaluated separately as FAQs and questions prepared by the NOGG. The scores given to the answers are expressed as percentages.

## Results

**Figure 1:**
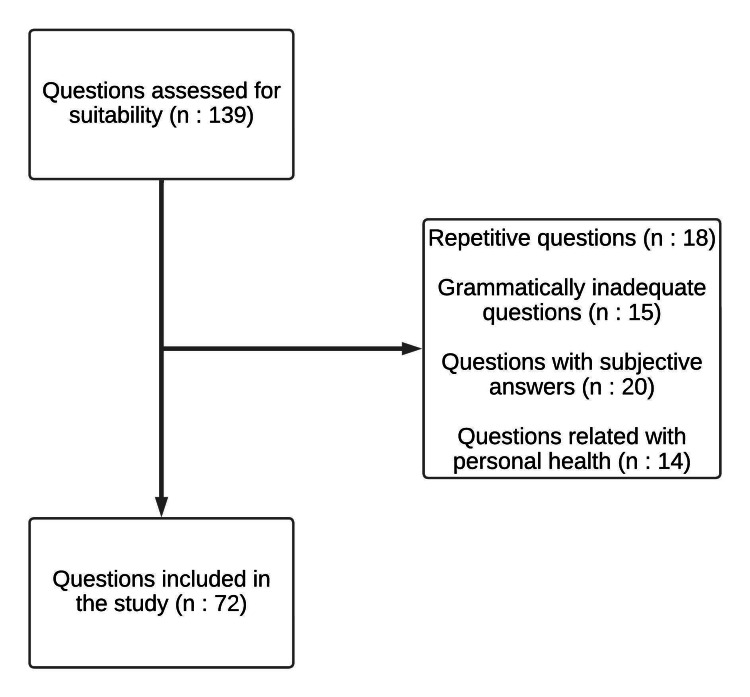
Flowchart of the study

The reasons why some questions about osteoporosis were not included in the study and the flow chart of the study were presented in Figure 1. While 139 number of questions were evaluated in total, it was determined that 67 of them did not meet the study criteria (repetitive questions, n = 18, grammatically inadequate questions, n = 15, questions with subjective answers, n = 20, and questions related with personal health, n = 14, respectively), and 72 questions were included in the study.

**Figure 2:**
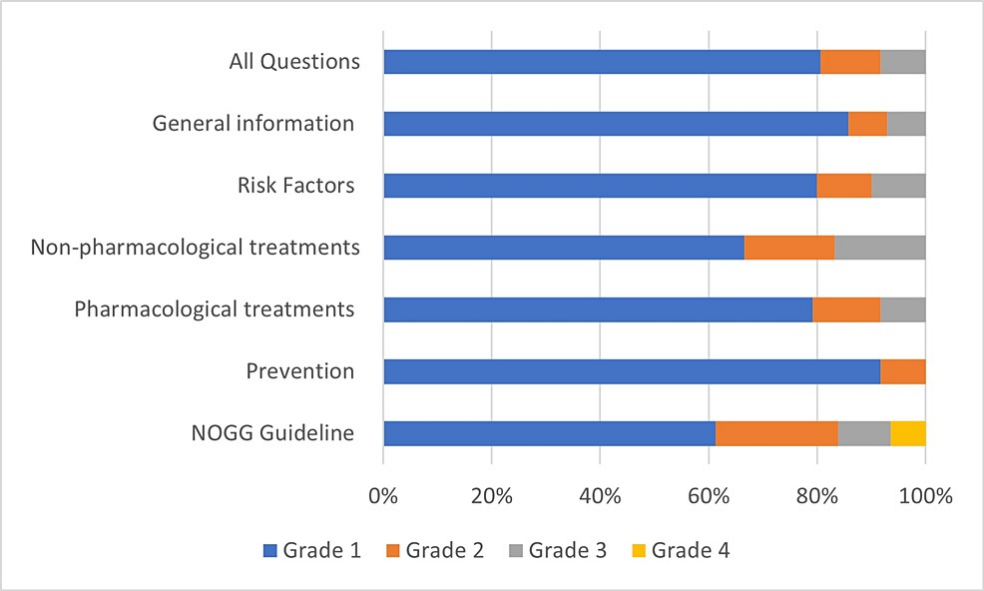
Grading the answers to the osteoporosis-related questions according to the question categories NOGG: National Osteoporosis Guideline Group

ChatGPT responded to 72 FAQ with an accuracy rate of 80.6% (58 questions). Total, eight (11.1%) answers given by ChatGPT were accepted as grade 2 and six (8.3%) answers as grade 3. None of the responses to the 72 FAQ were evaluated as category 4. The highest accuracy in ChatGPT's answers about osteoporosis was in the prevention category, 91.7%, and in the general knowledge category, 85.8%. Other side, the lowest accuracy rate (66.6%) was obtained in the responses related to non-pharmacological treatments. In addition, only 19 of the 31 (61.3%) questions prepared according to the NOGG guidelines were answered correctly by ChatGPT, and two answers (6.4%) were categorized as grade 4. The grading of responses to FAQs about osteoporosis and grading of answers to questions based on NOGG guidelines were documented in Table 1 and presented in Figure 2.

**Table 1:** Grading the answers to the osteoporosis-related questions according to the question categories NOGG: National Osteoporosis Guideline Group

	Grade 1	Grade 2	Grade 3	Grade 4
All Questions (n=72)	58 (80.6%)	8 (11.1%)	6 (8.3%)	
General information (n=14)	12 (85.8%)	1 (7.1%)	1 (7.1%)	-
Risk Factors (n=10)	8 (80.0%)	1 (10.0%)	1 (10.0%)	-
Non-pharmacological treatments (n=12)	8 (66.6%)	2 (16.7%)	2 (16.7%)	-
Pharmacological treatments (n=24)	19 (79.2%)	3 (12.5%)	2 (8.3%)	-
Prevention (n=12)	11 (91.7%)	1 (8.3%)	-	-
NOGG Guideline (n=31)	19 (61.3%)	7 (22.6%)	3 (9.7%)	2 (6.4%)

The reproducibility rate of ChatGPT answers on 72 FAQ was 86.1% and the reproducibility rate of ChatGPT answers on NOGG guidelines was 83.9%. The repeatability rate was highest in general information about osteoporosis (92.8%) answers and in risk factors (90.0%) responses (Table 2).

**Table 2:** The rate of giving the same answers for repetitive questions NOGG: National Osteoporosis Guideline Group

	Reproducibility, n (%)
All Questions (n=72)	63 (86.1%)
General information (n=14)	13 (92.8%)
Risk Factors (n=10)	9 (90.0%)
Non-pharmacological treatments (n=12)	10 (83.3%)
Pharmacological treatments (n=24)	21 (87.5%)
Prevention (n=12)	10 (83.3%)
NOGG Guideline (n=31)	26 (83.9%)

## Discussion

Artificial intelligence technology is rapidly entering our daily lives, and the opportunities brought by artificial intelligence are used more widely in the field of health. The correct integration of artificial intelligence in the field of health may enable the widespread and effective application of screening tests, and earlier diagnosis of diseases, and enable patients to be more compliant to follow-up schedules. Despite its advantages, there are many reservations about the use of artificial intelligence in the healthcare field [6-8]. Thus, the study aimed to clarify the knowledge of ChatGPT about osteoporosis, which affects almost one in six of the world's population. The present study revealed that ChatGPT gave completely correct answers for 80.6% of FAQs about osteoporosis. Additionally, the accuracy rate of ChatGPT responses was highest in questions about general information about osteoporosis and in questions about prevention of osteoporosis. Other side, ChatGPT answered completely true only 61.3% of the questions which were prepared according to NOGG guidelines. Lastly, reproducibility rates of ChatGPT answers were over 80% for both the FAQ and questions based on NOGG guidelines.

The accuracy and reliability of web sources about health topics are debatable, and numerous studies demonstrated that online content contains false and incomplete information. Alsyouf et al. analyzed contents of web sources including Facebook, Twitter, Instagram, etc., about prostate cancer, and the author stated that inaccurate information about prostate cancer in web resources was almost 30 times more than correct information [10]. In contrast, Caglar et al. analyzed the performance of ChatGPT’s answers about pediatric urology, and the author concluded that ChatGPT gave satisfactory answers to questions related to pediatric urology [7]. In another study, Bulck and Moons evaluated the responses of ChatGPT about cardiovascular diseases, and the authors stated that 17 out of 20 answers had a sufficient information for patients [11]. In the present study, the grade of answers by the ChatGPT to inquiries about osteoporosis was analyzed for the first time, and the findings of the study demonstrated that almost four of five ChatGPT responses about osteoporosis provided satisfactory and completely true information. Unlike other web resources, ChatGPT reviews multiple sources when answering questions, and extensive data usage may be associated with the high accuracy rate and qualification rate of ChatGPT's answers.

Guidelines are resources that make inferences based on the results of many meta-analyses and studies, have high scientific content, and contain information that is recommended to be performed in daily practice. Other side, questions about sources with evidence-based scientific literature may be more complicated to answer [12]. In a study by Antaki et al., ChatGPT answered the exam of first-year ophthalmology residents, and 55.8% of questions were answered completely true by ChatGPT, which was similar to residents’ results [13]. Also, Caglar et al. stated that ChatGPT accurately answered 93.6% of the questions based on pediatric urology guidelines [7]. However, the accuracy rate of ChatGPT answers to NOGG guidelines-based questions was 61.3% in the present study, which is much lower than the FAQ about osteoporosis.

Although the present study was the first to analyze the accuracy of ChatGPT responses in osteoporosis, this study has some limitations. The present study included a certain period, but contents about osteoporosis were incessantly uploaded to the internet. In addition, the study was done in only the English language. However, English is the most used language in the academic and social areas of the web. Additionally, responses were evaluated by a single clinician. Responses of ChatGPT about osteoporosis in rarer languages may be the subject of further studies. Lastly, we did not evaluate the intelligibility of ChatGPT responses, the intelligibility of ChatGPT by individuals with different educational levels could be analyzed in further research.

## Conclusions

Present study outcomes for the first time showed that ChatGPT provided adequate and sufficient answers to more than 80% of FAQs about osteoporosis. However, the accuracy of ChatGPT’s answers to inquiries based on NOGG guidelines was decreased to 61.3%. Our findings recommended that applying ChatGPT in fields of medicine dealing with osteoporosis will provide better information about osteoporosis to patients.

## Appendices

**Table 3:** Frequently asked questions about osteoporosis

General
What is osteoporosis, and how does it affect bone health?
What are fragility fractures, and why are they associated with osteoporosis?
Who is most at risk for developing osteoporosis and experiencing fragility fractures?
How is osteoporosis diagnosed, and what tests are commonly used to assess bone density?
What are the primary causes and risk factors for osteoporosis?
What lifestyle changes can help prevent or manage osteoporosis and reduce the risk of fractures?
What role does nutrition play in maintaining strong bones and preventing osteoporosis?
Can osteoporosis be hereditary, and how does genetics contribute to the risk of developing the condition?
What are some common signs and symptoms of osteoporosis, and how can they be recognized?
What medical treatments and medications are available for managing osteoporosis and reducing fracture risk?
How does physical activity and exercise impact bone health and help prevent fragility fractures?
Are there specific precautions that individuals with osteoporosis should take to avoid falls and fractures?
Can osteoporosis affect men, or is it primarily a condition that affects women?
What is the importance of early detection and treatment in preventing the progression of osteoporosis and reducing fractures?
Risk Factors
What are the main risk factors for developing osteoporosis?
How does age influence the risk of osteoporosis, and why is it more common in older adults?
What role does gender play in osteoporosis risk, and why are women more prone to the condition?
How does menopause impact a woman's risk of developing osteoporosis?
Can genetics and family history increase the likelihood of osteoporosis?
How does race and ethnicity affect the risk of osteoporosis?
What lifestyle factors, such as smoking and excessive alcohol consumption, contribute to osteoporosis risk?
How does a sedentary lifestyle increase the chances of developing osteoporosis?
Why is a history of previous fractures considered a risk factor for future fragility fractures?
How does long-term use of certain medications, like corticosteroids, influence the risk of osteoporosis?
Non-pharmacological treatments
What are nonpharmacological approaches to managing osteoporosis, and how effective are they compared to medications?
How does a balanced diet contribute to maintaining strong bones and preventing osteoporosis?
Can you provide dietary recommendations for calcium and vitamin D intake to support bone health?
What role does physical activity play in preventing and managing osteoporosis, and what types of exercises are recommended?
Are weight-bearing exercises more beneficial for bone health, and why?
How does maintaining a healthy body weight contribute to reducing the risk of osteoporosis?
What is the significance of avoiding smoking and excessive alcohol consumption in relation to bone health?
How does limiting caffeine intake impact bone health, and should individuals with osteoporosis avoid caffeine?
Can stress management techniques, such as meditation or yoga, have a positive impact on bone health?
How does getting adequate sleep contribute to bone health and overall well-being for those with osteoporosis?
What are some practical fall prevention strategies that can help individuals with osteoporosis avoid fractures?
Are there specific lifestyle changes or nonpharmacological interventions that postmenopausal women can adopt to reduce their osteoporosis risk?
Pharmacological treatments
What are bisphosphonates, and how do they work to treat osteoporosis?
What are some common bisphosphonate medications used for osteoporosis treatment?
How are bisphosphonates taken, and what are the dosing schedules?
What are the potential side effects and risks associated with bisphosphonate use?
How long is bisphosphonate treatment typically recommended, and is there a maximum duration of use?
What are SERMs, and how do they help in treating osteoporosis?
Can you provide examples of SERMs commonly used for osteoporosis treatment?
Are there any specific populations for whom SERMs are particularly beneficial or not recommended?
What is denosumab, and how does it work to treat osteoporosis?
How is denosumab administered, and what is the dosing schedule?
What are the potential benefits and risks associated with denosumab treatment?
What are teriparatide and abaloparatide, and how do they differ from other osteoporosis medications?
How are these medications administered, and what is their mechanism of action?
Who is typically considered a candidate for teriparatide or abaloparatide treatment?
What is calcitonin, and how does it work to treat osteoporosis?
Is calcitonin still commonly used for osteoporosis treatment, or have other medications become more preferred?
Are there instances where combining different osteoporosis medications might be recommended?
How are combination therapies typically chosen, and what benefits can they offer?
How often should bone density be monitored during osteoporosis treatment?
What other tests or assessments are important to track the effectiveness of pharmacological treatment?
Can osteoporosis medications be used long-term, or are there considerations for discontinuation?
What factors might influence the decision to discontinue osteoporosis treatment?
Are there specific recommendations for osteoporosis treatment in postmenopausal women, men, and individuals with certain medical conditions?
How does a healthcare professional determine the most appropriate pharmacological treatment for an individual's osteoporosis?
Prevention
What are the key steps individuals can take to prevent the development of osteoporosis?
How does a balanced diet contribute to preventing osteoporosis, and which nutrients are particularly important for bone health?
What role does calcium play in preventing osteoporosis, and what are good dietary sources of calcium?
How does vitamin D contribute to bone health and osteoporosis prevention, and what are sources of vitamin D?
What is the recommended amount of weight-bearing exercise for maintaining strong bones and preventing osteoporosis?
Can you suggest specific weight-bearing exercises that are effective in preventing osteoporosis?
How does avoiding smoking and excessive alcohol consumption contribute to osteoporosis prevention?
What role does caffeine intake play in bone health, and should individuals limit their consumption to prevent osteoporosis?
Are there specific age-related considerations for preventing osteoporosis, particularly in postmenopausal women and older adults?
What lifestyle changes can young adults make to reduce their risk of developing osteoporosis later in life?
Are there certain medical conditions or medications that might increase the risk of osteoporosis, and how can they be managed to prevent bone loss?
How does a history of fractures or family history of osteoporosis influence preventive measures that individuals should take?

**Table 4:** Guideline recommendations

Guideline Recommendations
1. What screening test should be performed on people at moderate risk of fracture to improve the 10-year risk estimate?
2. What testing should be done in individuals at high and very high risk of fractures to guide drug selection and provide a basis for BMD monitoring?
3. In which patient group with acute-onset back pain, vertebral fracture should be considered in the foreground?
4. What kind of treatment should be applied to patients with osteoporosis and/or fragility fractures?
5. In which risk group should drug therapy be started for osteoporosis?
6. If BMD measurement is impractical (eg due to frailty), what should be done to guide treatment decisions?
7. What kind of treatment is applied in osteoporosis patients, especially in elderly people, with previous and/or new fragility fractures?
8. What factors should be considered when starting treatment for osteoporosis?
9. How long after fragility fracture should treatment be started? From where?
10. What are the indications for referral of very high risk osteoporosis patients to a secondary care center?
11. Which oral and iv treatments are recommended in the first step in patients with osteoporosis treatment indications?
12. What are the alternative treatment options if primary-line bisphosphonates are not suitable or tolerated?
13. What treatments should be followed following treatment with teriparatide or romosozumab?
14. Should patients' tolerance and compliance with oral drug treatments be monitored?
15. Why is osteoporosis treatment long-term?
16. How long should oral bisphosphonates be given in the treatment of osteoporosis?
17. What should be considered before starting denosumab treatment?
18. In which situations should denosumab treatment be discontinued?
19. How often must the fracture have occurred to repeat the fracture risk assessment after each new fracture?
20. How long after discontinuing drug therapy should he reassess fracture risk?
21. Which treatments should be started without waiting for DXA screening in patients at risk for osteoporosis?
22. Which type of treatment should be planned in patients with a very high risk of vertebral fracture?
23. Which alternative treatments should be considered in patients at risk for osteoporosis?
24. What lifestyle changes should be recommended to patients in order to prevent osteoporosis?
25. Which foods should be recommended for patients to consume in order to prevent osteoporosis?
26. What kind of exercises should be recommended to patients in order to prevent osteoporosis?
27. How should patients with fragility fractures be followed up?
28. In which patient group is it necessary to routinely evaluate the spine in all imaging?
29. For which muscle groups should exercise programs be applied for patients at risk for osteoporosis?
30. What should be done for a patient who develops a fragility fracture?
31. What would it take to reduce the risk of further fractures?
